# Energy-Efficient geopolymer wall panels: optimizing mechanical, thermal, and acoustic properties for sustainable construction

**DOI:** 10.1038/s41598-025-11783-4

**Published:** 2025-07-16

**Authors:** Nidhya Rathinavel, Abdul Aleem, Mohamed Ismail

**Affiliations:** Engineering Materials Laboratory, Department of Civil Engineering, PSG Institute of Technology and Applied Research, Neelambur, Coimbatore, 641 062 India

**Keywords:** Geopolymer; waste material utilization, Thermal performance, Acoustic performance, Noise reduction coefficient, Environmental impact, Civil engineering

## Abstract

The rising demand for sustainable and multifunctional building materials in advanced construction field needs innovative composites that acquires superior mechanical, thermal, and acoustic performance. This study focusses on to analyse the potential of geopolymer composites developed with ground granulated blast furnace slag (GGBS), waste foundry sand (WFS), vermiculite, and coir fiber. Five mix designs (M1–M5) were developed with varying combination of WFS (5–25wt%) and vermiculite content (40–20wt%), while maintaining constant liquid-to-binder ratio (L/B = 0.4) and coir fiber content (5wt%). The test findings highlight, impact of the mix design on material performance. In this study, for the sample M1 to M5, densities varied from 1302 to 2032 kg/m³, water absorption reduced from 23 to 9%, compressive strength improved from 6.52 MPa to 20.0 MPa, while flexural strength increased from 2.9 MPa to 7.0 MPa, indicating improved structural integrity with increasing WFS. In addition, thermal conductivity varied from 0.1222 to 0.1652 W/m·K, and sound absorption coefficients (SAC) peaking at 0.41, while noise reduction coefficients (NRC) ranged from 0.23 to 0.10, demonstrating superior thermal and acoustic performance in mixes with higher porosity due to vermiculite. Further, the study emphasis the versatility of geopolymer composites, which can be tailored for specific applications by adjusting the mix proportions. The test findings position geopolymer composites as a promising solution for sustainable, multifunctional building materials in modern construction.

## Introduction

The construction industry primarily faces a dual challenge such as accelerating urbanisation and mitigating environmental impacts. Urbanization has intensified the demand for infrastructure, leading to the depletion of natural resources and an increase in greenhouse gas emissions. Simultaneously, environmental sustainability has become a pressing concern, necessitating the development of eco-friendly materials and practices to mitigate the sector’s carbon footprint. As the global population expected to cross over 9 billion by 2050, urban areas are forecasted to build house around 68% of the world’s population, driving unprecedented demand for sustainable infrastructure^1^. Despite this, Portland cement, a cornerstone of modern construction, is a significant contributor to global CO₂ emissions, accounting for approximately 7–8% of total emissions, which generates 0.9 tons of CO₂ for every ton of cement production^2,3^. Its energy-intensive manufacturing process and reliance on natural limestone have profound environmental implications, underscoring the urgent need for sustainable alternatives. These parameters emphasise the pressing need for novel, energy-efficient, and environmentally sustainable construction materials^3^.

Geopolymer technology has recognised as a viable alternative to conventional construction technology. Geopolymer facilitates dual benefits of lowering carbon footprint and providing circular economy by integrating industrial by products like fly ash, GGBS, and WFS^4–6^. In contrast to Portland cement, geopolymers are formulated by an alkali-activation process, which transforms aluminosilicate precursors into a stable, hardened composite. This method needs notably lowers input energy and promotes up to reduction of 80% CO₂ emissions, facilitating geopolymers a crucial component in the production of green construction materials^5,7^.

Geopolymers are synthesized through the alkaline activation of aluminosilicate materials. Ground Granulated Blast-Furnace Slag (GGBS) and Waste Foundry Sand (WFS), metallurgical by-products, play a pivotal role in geopolymerization. The chemical reaction forms calcium-aluminosilicate-hydrate (C-A-S-H) and sodium-aluminosilicate-hydrate (N-A-S-H) gels, which are responsible for the high strength and durability of geopolymer materials. Recent studies have demonstrated the potential of incorporating granite waste powder and other by-products to enhance the mechanical properties of geopolymers across varying molarities of activators^8–11^. In addition, the importance of alkaline motion in geopolymer systems extends beyond strength enhancement; it reduces the carbon footprint by enabling the use of industrial waste materials. Furthermore, the integration of natural fibers into geopolymers has shown promise in improving tensile strength, crack resistance, and overall performance, while simultaneously reducing material rejection and waste^10,11^.

While considering this, the possibility of combining different industrial by-products with natural additives to tailor geopolymer performance for specific purposes has been investigated recently. Due to its resilient reactivity, GGBS has been demonstrated to significantly boost the initial strength of geopolymer concrete. Research indicates that when GGBS is incorporated with other substances, its compressible strength can be enhanced to 80.2 MPa^12^. WFS serves as an excellent filler that promotes waste management efforts while improving mechanical performance. It is a useful resource in geopolymer compositions because its incorporation improves strength and durability^13^. Vermiculite offers both thermal and acoustic insulation due to its low density and porous nature. Due to this attribute, it is a perfect addition to environmentally friendly building panels, promoting environmentally friendly building methods^14^. Coir and additional natural fibers are incorporated into geopolymer composites to improve their resistance to damage. These cellulose fibers increase the material’s overall strength as well as environmental responsibility through reduced shrinkage and cracking^15^.

According to studies, fiber reinforced geopolymer wall panels are appropriate for sustainable building construction as it possesses high thermal and acoustic properties. The integration of natural fibers like jute, cotton, bamboo, coir are significantly improving the thermal and acoustic insulation property. With thermally conductivity values as low as 0.316 W/m·K, geopolymer composite enriched with biological waste materials and incorporating melamine fibers offer exceptional thermal insulation^14,16^. Because natural fibers increase thermal resistance and decrease thermal conductivity, geopolymer materials are a viable option for energy-efficient construction^17^. These composites exhibit excellent acoustic insulation characteristics, and noise absorption values that reach up to 0.70 at 500 Hz^16^. The inclusion of organic fibers optimizes noise absorption even further, particularly by lowering impact sound propagation at low frequencies. As Geopolymers exhibit superior thermal insulation and acoustic properties, as evidenced by prior research, making them suitable for energy-efficient and noise-reducing applications in construction. These attributes, combined with their cost efficiency and reliance on industrial byproducts, position geopolymers as a sustainable alternative capable of achieving sectoral economies^18^. Further, from the previous studies, the thermal and acoustic functionalities of geopolymer wall panels is potentially enhanced by various modifications and material mix designs. These studies underline the suitability of geopolymer materials for energy-efficient and noise-reducing construction applications, further promoting their adoption in the sector^19^. Despite fiber reinforcing increases thermal and acoustic characteristics, additional study needs to be done for ensuring the materials’ longevity in an array of building scenarios because of their impact on the environment and long-term durability.

In addition to sustainability, geopolymers promotes excellent functional properties that mitigate to the needs of advanced energy-efficient construction. For instance, Geopolymer foam composites integrating silica aerogel attain a least thermal conductivity of 0.133 W/(m·K), exhibiting exceptional thermal insulation potentials^20^. The addition of huge silica aerogel particles in geopolymer foam improves sound absorption, with a mean absorption coefficient of 0.51^20^, while the design of fiber-reinforced panels, which integrate high sound-absorbing layers, leads to effective sound insulation^21^. Further, previous study shows that geopolymer panels exhibit acoustic absorption coefficients ranging from 0.7 to 1.0 at lower frequencies (40–150 Hz) and 0.1 to 0.3 at higher frequencies (800–1600 Hz)^21^. The integration of fibres content further improves acoustic absorption, with minimal dosage attaining a mean coefficient of 0.51^20^.

As the construction industry has increasingly embraced geopolymers as sustainable alternatives to traditional cement-based materials, yet their application in structural elements like wall panels remains underexplored. Research gaps include limited focus on wall panels, with minimal attention to unique challenges such as load-bearing capacity, thermal insulation, and noise reduction; insufficient investigation of performance under realistic environmental conditions, including temperature variations, humidity, and long-term durability^22^; and a lack of studies on integrating industrial byproducts like GGBS and WFS to enhance performance. Additionally, existing research often isolates properties like compressive strength, underscoring the need for holistic evaluations of mechanical, thermal, and acoustic performance^23^. This study addresses these gaps by proposing a novel geopolymer mix design incorporating GGBS and WFS to ensure strength, durability, and sustainability; evaluating multi-functional performance under laboratory and simulated conditions; emphasizing sustainability through the use of industrial byproducts to reduce carbon footprints; and bridging the gap between laboratory research and real-world applications. These efforts aim to advance the understanding of geopolymer wall panels and their transformative potential for sustainable construction.

This research primarily focuses on developing and evaluating energy-efficient geopolymer wall panels by systematically varying the mix design. In this study, five geopolymer mix designs were developed, varying the proportions of WFS and vermiculite while retaining GGBS and coir fiber constant. Key parameters evaluated include:

- Density: Influences the structural stability and weight of the material, directly impacting transportation and installation energy requirements.
- Water Absorption: Indicates the porosity and durability of the material, essential for assessing its resistance to environmental degradation.
- Compressive and Flexural Strength: Measures the load-bearing capacity and mechanical robustness, critical for structural applications.
- Thermal Conductivity and Resistance: Determines the material’s insulation capabilities, addressing the need for energy-efficient construction.
- Acoustic Performance: Assessed through the Sound Absorption Coefficient and Noise Reduction Coefficient (NRC), these parameters contribute to occupant comfort and noise mitigation.

The findings of this study demonstrate the potential of geopolymer composites as multifunctional construction materials that meet the growing demands for sustainable and energy-efficient infrastructure. Compared to traditional materials, the developed geopolymer wall panels exhibit superior thermal insulation, acoustic performance, and mechanical properties, offering a versatile solution for modern construction challenges. In addition to its technical contributions, this research aligns with global sustainability goals by emphasizing resource efficiency and waste valorization. By incorporating industrial by-products and renewable natural fibers, the study supports efforts to minimize landfill waste, reduce carbon footprints, and conserve natural resources. The results highlight the scalability of geopolymer technology from laboratory research to practical applications, showcasing its transformative potential in the construction industry.

## Experimental methods

### Raw materials

The materials used in this study were selected for their complementary roles in enhancing the thermal and acoustic properties of geopolymer wall panels. The raw materials like GGBS was procured from a RR Enterprices, Chennai, is a byproduct of the blast furnace process used in ironmaking. GGBS served as the primary binder due to its excellent reactivity and contribution to strength development through geopolymerization. Another material, WFS sourced from local steel casting industry, was incorporated as a filler to improve the density and mechanical strength of the mix, leveraging its uniform particle size and inert characteristics. Vermiculite sourced from commercial supplier, a lightweight material with excellent thermal insulation properties, was added to reduce thermal conductivity. Vermiculite is incorporated into the geopolymer mix to enhance thermal insulation. Its layered structure and low thermal conductivity significantly reduce heat transfer through the material, making it suitable for applications requiring energy-efficient construction materials^24^. Table 1 displays the particle size distribution and Table 2 displays mineral composition of GGBS, vermiculate and WFS. The particle size distribution and chemical composition of the materials used in this study significantly influence their roles and effectiveness in geopolymer applications. GGBS with a particle size ranging from 1 to 100 microns and a median size (D50) between 10 and 30 microns, is finely ground to achieve specific surface area of 500–700 m²/kg^25^. This fine particle size improves its reactivity, making it highly applicable for cementitious reactions and geopolymerization. Chemically, GGBS contains 30–50% silica (SiO₂), 6–13% alumina (Al₂O₃), and 30–50% calcium oxide (CaO), which collectively contribute to its pozzolanic and latent hydraulic properties. Minor components include magnesium oxide (2–10%) and trace amounts of other compounds like SO₃ and TiO₂.

**Table 1:** Particle size Distribution.

Material	Particle Size Range (µm)	Median Particle Size (D50, µm)	Typical Fineness/Specific Surface Area	Remarks
**GGBS**	1–100	10–30	500–700 m²/kg	Fine particles suitable for cementitious reactions.
**Vermiculite**	50–2000	250–500	Not applicable	Larger particles for thermal insulation; finer grades for lightweight concrete.
**WFS**	75–1000	250–600	Not applicable	Uniform particle size, suitable as sand replacement.

Vermiculite, a phyllosilicate mineral known for its lamellar structure, exhibits a broader particle size range of 50 to 2000 microns, with median sizes of 250–500 microns depending on the grade. Coarser particles are typically used for thermal insulation, while finer grades are incorporated into lightweight concrete. Unlike GGBS, vermiculite lacks a standard specific surface area metric, as its application depends on its physical properties rather than reactivity. Chemically, vermiculite is composed of 30–40% silica, 15–10% alumina, and 25–35% magnesium oxide (MgO), contributing to its thermal insulation properties. It also contains iron oxide (15–25%) and potassium oxide (6–12%), along with trace impurities such as quartz and feldspar.

WFS, features a particle size range of 65 to 1000 microns, with most particles falling within 250–600 microns. This uniform particle size makes WFS a viable replacement for natural sand in construction. Chemically, WFS is primarily composed of silica (60–90%) with smaller amounts of alumina (2–8%), iron oxide (1–6%), and other minor oxides. Depending on its previous use, WFS may also contain organic binders or impurities that require treatment before incorporation into construction materials^26^.

Coir fibers, sourced from coconut husks, were used as additives to improve flexural strength and crack resistance, owing to their high tensile strength and natural bonding with the geopolymer matrix. Additional materials included NaOH pellets (96% purity), waterglass (64.5 wt% H_2_O, 26.98 wt% SiO_2_, and 8.53 wt% Na_2_O), and tap water^27^. Prior to experiments, NaOH pellets, waterglass, and tap water were mixed well and cooled to produce the alkaline activator. The alkali activator consisted of a sodium silicate to sodium hydroxide solution (10 M concentration) in a 2.5:1 ratio, facilitating the geopolymerization reaction by activating the GGBS binder^28–30^. The careful selection and characterization of these materials ensure their complementary roles in enhancing the mechanical, thermal, and acoustic performance of geopolymer-based systems. GGBS contributes to strength and reactivity, vermiculite provides thermal insulation, and WFS acts as a sustainable alternative to natural sand, improving density and flowability.

**Table 2:** Chemical Composition Table

Component	GGBS (%)	Vermiculite (%)	WFS (%)
**SiO₂ (Silica)**	30–50	30–40	60–90
**Al₂O₃ (Alumina)**	6–13	15–20	2–8
**CaO (Calcium Oxide)**	30–50	1–3	< 1
**MgO (Magnesium Oxide)**	2–10	25–35	0.5–2
**Fe₂O₃ (Iron Oxide)**	0.5–2	15–25	1–6
**K₂O (Potassium Oxide)**	< 1	6–12	0.5–2
**Other Compounds**	Trace amounts of SO₃, TiO₂, and MnO	Trace elements and water content	Organic binders or impurities (depending on binder).

### Composite Preparation

The composites with five mix designs (M1 to M5) were formulated, varying the proportions of WFS and vermiculite while keeping GGBS and coir fiber content constant at 55% and 5% by weight, respectively. The mix design developed is detailed in Table 3. The mortars had a L/B ratio of 0.40, an alkali dosage (the Na_2_O-to-binder ratio) of 6.0 wt%, a silicate modulus (the SiO_2_-to-Na_2_O molar ratio of the alkaline activator) of 1.15. A liquid-binder ratio of 0.4 was chosen based on prior studies to ensure adequate workability and sufficient binder activation for geopolymerization^31^. This ratio balances the flowability of the mix with its setting and mechanical properties^32,33^. The specific proportions were adapted to evaluate the effects of increasing WFS content and decreasing vermiculite content on the thermal, acoustic, and mechanical performance of the geopolymer panels.

**Table 3:** Mix design.

Mix ID	GGBS (wt%)	Foundry Sand (wt%)	Vermiculate (wt%)	Coir fiber (additive) %	Liquid to binder ratio	Curing
M1	55	5	40	5	0.4	Ambient condition
M2	55	10	35	5	0.4	Ambient condition
M3	55	15	30	5	0.4	Ambient condition
M4	55	20	25	5	0.4	Ambient condition
M5	55	25	20	5	0.4	Ambient condition

Initially, the dry components (GGBS, WFS, and vermiculite) are mixed in a mechanical mixer. The dry materials should be blended evenly for 3–5 min to prevent segregation and ensure uniformity^34^. In parallel, the alkali activator solution is prepared by dissolving sodium hydroxide (NaOH) in water to achieve an 10 M concentration. This is then mixed with sodium silicate solution in a ratio of 2.5:1 (Na₂SiO₃: NaOH) to create a highly alkaline solution that activates the GGBS and foundry sand^35^. The alkali solution is allowed to cool to room temperature before being added to the dry mix. The prepared alkali activator is slowly added to the dry mixture while continuously mixing. The liquid to binder ratio is maintained at 0.4 to ensure proper activation of the binder materials. The mixture is stirred thoroughly until a homogeneous paste is achieved. The coir fibers are pre-soaked in water for 30 min to enhance their dispersion within the mix and ensure better bonding with the binder materials. This soaking process enhances the bond strength and overall durability of the composite^36^. Gradually, the pre-soaked coir fiber is added into the homogeneous paste. The fibers must be uniformly distributed to prevent clumping and ensure consistent reinforcement throughout the composite material.

**Fig. 1:**
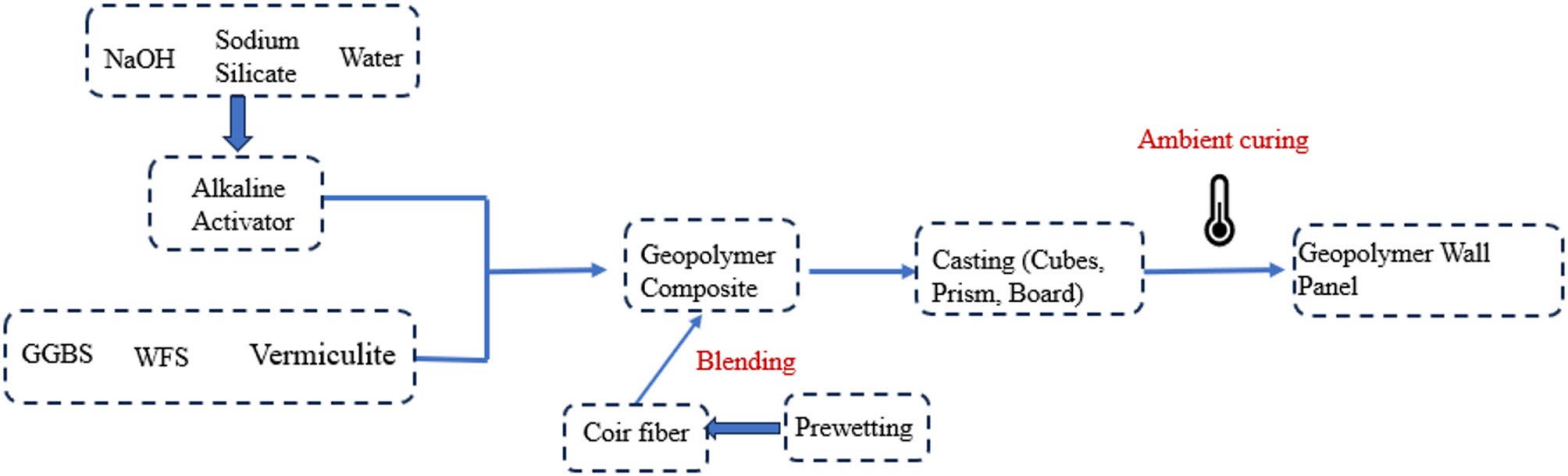
Process of making geopolymer wall panel^37^.

The development of geopolymer wall panels involved a streamlined process comprising the preparation of raw materials (GGBS, WFS, and vermiculite), the preparation of the alkaline solution, mixing of dry and wet components, casting samples into molds, curing at ambient temperatures, and testing for mechanical, thermal, and noise properties. This systematic approach ensures reproducibility and scalability, making the panels viable for practical applications. The process flow of geopolymer composite wall panel development is illustrated in Fig. 1.

The mixed material is poured into molds of the required shape and size, such as 50 mm × 50 mm × 50 mm cubes for compressive strength testing, prism molds (160 mm x 40 mm x 40 mm) for flexural testing and 300 mm x 300 mm x 15 mm particle board for thermal and acoustic testing. The molds should be pre-oiled or lined to facilitate easy demolding after curing. The mixed material is compacted in the molds to eliminate air pockets and ensure a dense, homogeneous specimen^38–40^. The specimens were cured for 28 days under controlled conditions to ensure consistency in the geopolymerization process. Specifically, the specimens were cured at a constant temperature of 25 °C (± 2 °C) and a relative humidity of 90% (± 5%). To maintain these conditions, the specimens were stored in a sealed curing chamber equipped with humidity control to prevent moisture loss during the curing period. These curing parameters were selected based on standard practices in geopolymer research and to simulate ambient curing conditions typically encountered in construction applications. While elevated-temperature curing (typically 60–90 °C for 24–48 h) is widely used in laboratory studies to accelerate geopolymerization and enhance early-age strength, ambient curing has gained attention for its applicability in real-world scenarios, especially for large-scale construction where energy-intensive curing is impractical. Several recent studies^41^ have demonstrated the viability of ambient curing for geopolymer systems incorporating GGBS and other reactive precursors, as these materials provide sufficient calcium and aluminosilicate content to facilitate geopolymerization at room temperature. After the curing period, the specimens are then stored under controlled conditions (room temperature with 50% relative humidity) until testing.

### Methods of testing

In this study, density and water absorption, compressive strength, and flexural strength of five types of fibre integrated geopolymer insulation wall panel were investigated. In addition, the thermal characteristics and acoustic performance of geopolymer wall panels were recorded. To better explore the surface and cross-section characteristics of the samples, microscopic morphology was observed.

#### Dry density and water absorption

The dry density of each cubic sample (50 × 50 × 50 mm³) was assessed to determine the overall density of the material, following ASTM C138^27^. Prior to testing, all samples were pre-dried in an oven at 60 °C until a constant mass was achieved. The mass of each sample was then measured to two decimal places. Equation 1provides the formula for calculating the bulk density:

1 $$\:\rho\:=m/V$$

where m represents the mass of the dry sample (kg), and V represents the volume of the sample (m^3^).

Compliance tests on water absorption of geopolymer composites were carried out in accordance with ASTM C1185^42^ and C1186^43^. Water absorption is a routine test with relative values. The test is made to determine the tendency of a product to absorb water and sometimes determine the uniformity of the product. Adhering to ASTM standards ensures that our findings are comparable with existing literature and meet the rigor necessary for scientific validation. Additionally, using these standards provides a framework for replicating our results, thereby enhancing the applicability of our research for both academic and industrial purposes. The rectangular bar samples were used as representative segments of plates. Water absorption (WA) was calculated using the following equation Eq. 2:

2 $$\:WA=\left(\frac{{W}_{s}-{W}_{d}}{{W}_{d}}\right)x\:100,\:mass\:\%$$

Where:

$\:{W}_{s}$ = saturated mass (g) of specimen submerged for 48 ± 8 h,

$\:{W}_{d}$ = dry mass (g) of specimen

#### Compressive strength

The geopolymer composites underwent compressive strength testing according to ASTM C109^44^. Cubic specimens measuring 50 × 50 × 50 mm was cured for 28 days. The compression test was conducted using a universal testing machine with a 2000 kN capacity and a constant displacement rate of 2 mm/min. The high precision of the UTM ensures accurate and reproducible results, critical for assessing the material’s mechanical performance. The compressive strength of each cubic sample was determined by averaging the results obtained from three specimens. Three specimen replicates were tested for strength. The compressive strength is calculated using the following formula Eq. (3):

3 $$\rm{P=F/A}$$

where, *P* is the compressive strength (MPa), *F* is the maximum load applied on the sample (kN), and *A* is the cross-section area of the sample (m^2^).

#### Thermal conductivity

After curing, a hardened specimen was split in half and dried at a temperature of 60 °C until its mass stabilized. Then, the specimen was immediately vacuum-packed and cooled down to room temperature. The thermal conductivity of the specimen was measured according to the standard ASTM D 5930 (2017) using the Xiatech TC3100 instrument (0.001–20 W/(m∙K)) through the transient hotwire method. Each specimen was tested five times for required accuracy, and the test chamber was maintained at a temperature of 25–27 °C and relative humidity of 45–50%^45,46^.

#### Sound absorption coefficient

The sound absorption coefficient of geopolymer composites was tested using the impedance tube method. The principle was based on the transfer function measurements, as described in the GB/T 18696.2–2002 standard^47^. During the test, the sample was placed in one end of the impedance tube and the other end is the sound source. Two microphones were placed along the tube, and the incident and the reflected sound pressure were measured separately, while their transfer functions were obtained. According to the transfer function, the sound reflection factor (r) can be calculated. Equation 4 provides the formula for calculating the sound absorption coefficient:

4 $$a=1-|r|^2$$

**Fig. 2:**
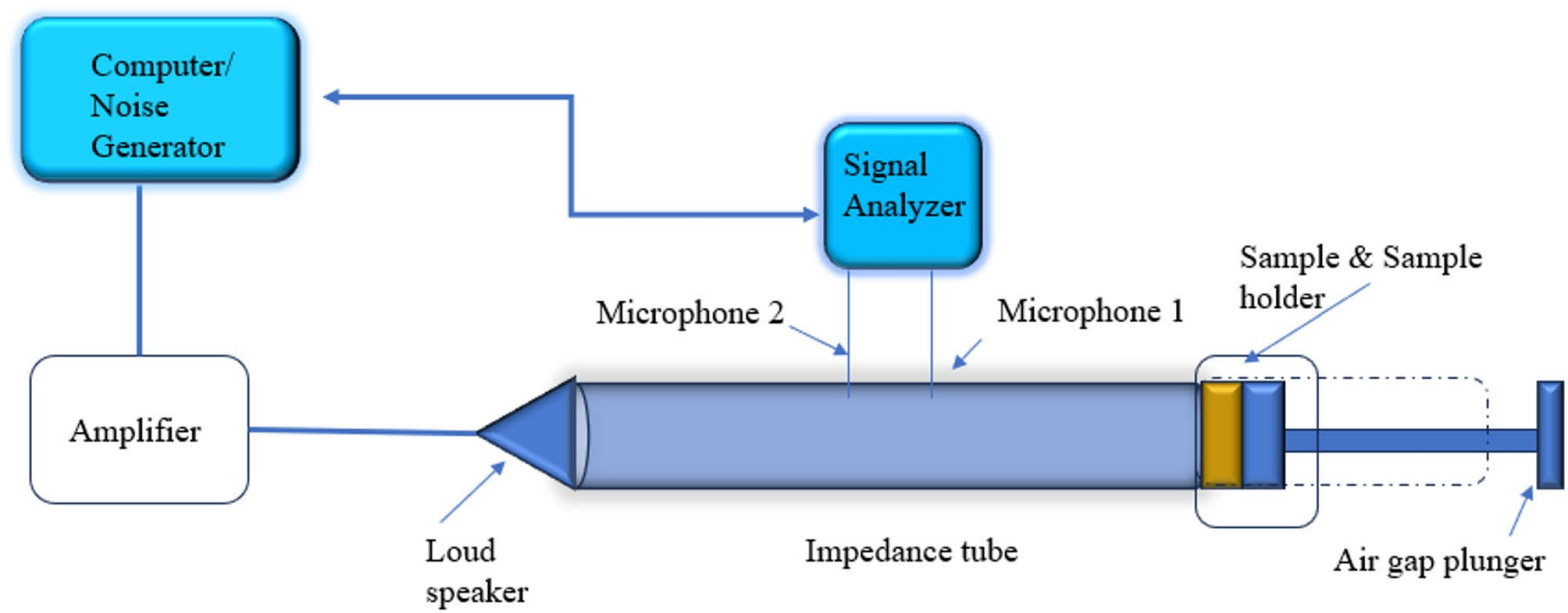
Sound Absorption impedance tube experimental setup.

**Fig. 3:**
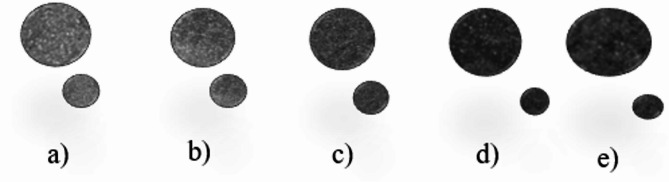
The samples for acoustic absorption (**a**). Sample M1, (**b**). Sample M2, (**c**). Sample M3, (**d**). Sample M4, (**e**). Sample M5^37^.

The impedance tube (BK 4206) used in the experiment was shown in Fig. 2. And the sample (see illustration of Fig. 3) with a diameter of 99 mm and a height of 15 mm was prepared according to the test requirements. The sample surfaces are polished before starting of the test. Due to the limitation of the fixed diameter of the impedance tube, the frequency range of the test was from 125 Hz to 6300 Hz. Every sample was tested twice to ensure the reliability of the results.

#### Noise reduction coefficient (NRC)

The NRC was determined to evaluate the composites sound absorption properties further. NRC provides an indicator of sound absorption effectiveness by averaging the SAC ($\:\alpha\:)$values at specific frequencies (250 Hz, 500 Hz, 1000 Hz, and 2000 Hz) as outlined in ASTM C423^42^. This allows a comparison of the overall sound absorption performance of different materials. Equation 5 provides the formula for calculating the sound absorption coefficient^27^:

5 $$\:NRC=({\alpha\:}_{250}+{\alpha\:}_{500}+{\alpha\:}_{1000}+{\alpha\:}_{2000})/4$$.

#### Microscopic morphology

Morphological observations were performed on the surface and cross-section of the materials by a Field Emission Scanning Electron Microscopy (FESEM). In addition, the samples were cut into small pieces and the surface was trimmed and polished before observation.

## Results and discussion

### Dry density and water absorption

**Fig. 4:**
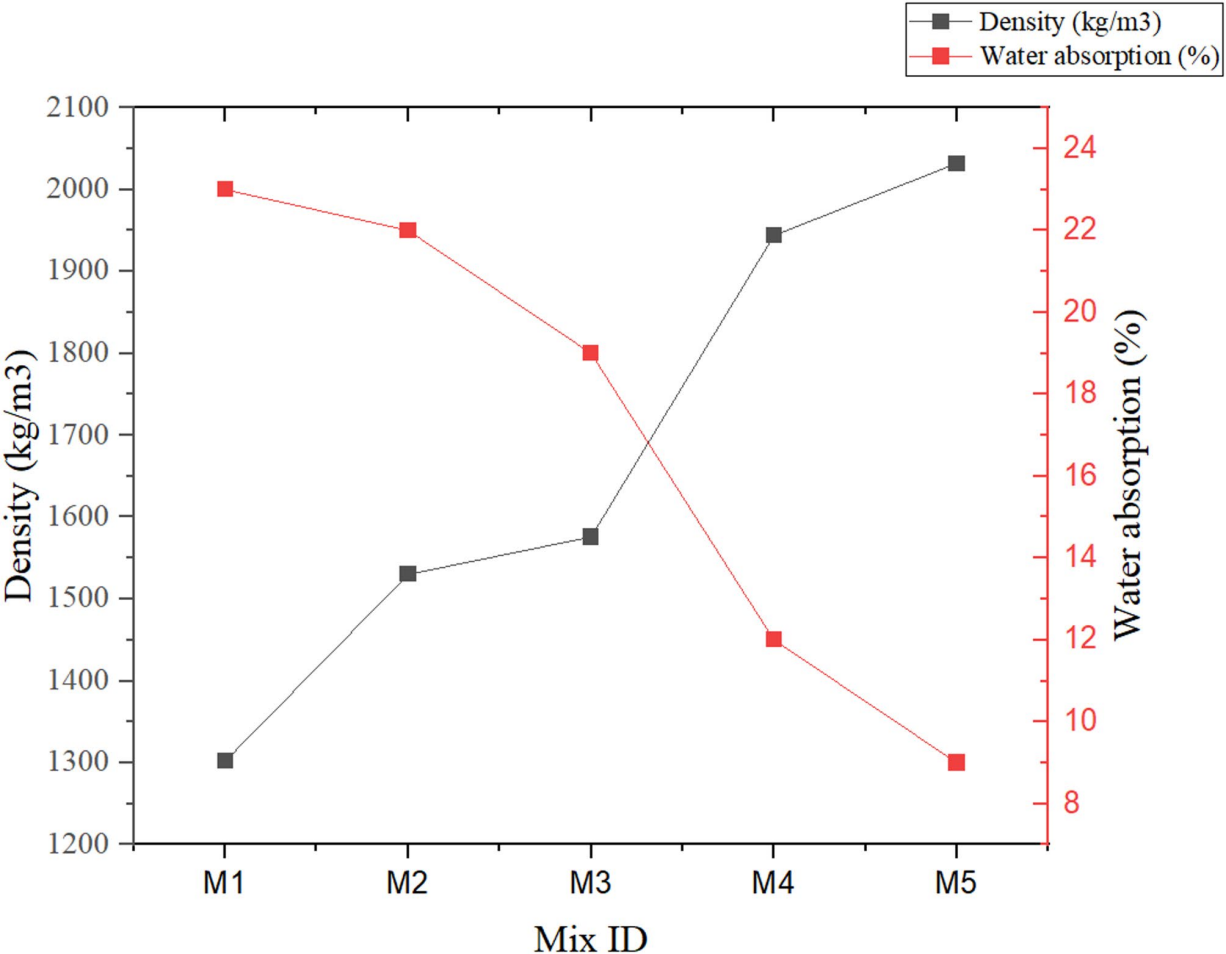
Dry density and Water absorption.

The density of the mixes reveals a clear trend influenced by the material composition, particularly the proportions of vermiculite and WFS. Figure 4 illustrates the density vs. water absorption trends. As the vermiculite content decreases and the foundry sand content increases, the density of the mixes rises significantly. M1, with the maximum vermiculite content (40%) and the lowest WFS content (5%), shows the lowest density of 1302 kg/m³. The lightweight and porous nature of vermiculite significantly lowers the overall density of the mix. As WFS replaces vermiculite in subsequent mixes, the density gradually increases, reflecting the higher bulk density of foundry sand compared to vermiculite. M2 and M3, with intermediate combination of vermiculite (35% and 30%, respectively) and WFS (10% and 15%, respectively), demonstrate medium densities of 1530 kg/m³ and 1576 kg/m³. This gradual increase highlights the densifying effect of substituting vermiculite with foundry sand, as the mix becomes less porous and more compact^28^. The highest densities are observed in M4 (1944 kg/m³) and M5 (2032 kg/m³), where vermiculite content is at its lowest (25% and 20%, respectively) and foundry sand content is at its highest (20% and 25%, respectively)^48^. The sharp increase in density in these mixes underscores the dominance of foundry sand’s denser structure, which outweighs the lightweight contribution of vermiculite. These findings are critical for determining the suitability of the mixes for various applications. M1 and M2, with lower densities, are ideal for lightweight applications, such as non-structural panels or insulation layers where reducing dead load is a priority^49^. Conversely, M4 and M5, with their higher densities, are better suited for structural applications or areas requiring higher mechanical strength, as the increased density correlates with improved load-bearing capacity^48^.

The water absorption characteristics of the mixes demonstrate a significant dependence on the material composition, particularly the balance between vermiculite and WFS. As the proportion of vermiculite decreases and the WFS content increases, water absorption reduces notably. The sample M1, with the maximum vermiculite content (40%), shows the highest water absorption rate at 23%. Highly porous nature of vermiculite enables the composite tends to retain the water. In addition, hydrophilic nature of vermiculite also leads to its high-water absorption capacity, enabling mixes with a higher proportion of this material more prone to moisture retention. As the vermiculite content is gradually reduced in M2 (35%) and M3 (30%), and foundry sand content increases to 10% and 15%, respectively, water absorption decreases to 22% and 19%. This trend reflects the reduced porosity and higher compactness of the mixes, as foundry sand, being less porous, limits the overall capacity for water retention. The lowest water absorption values are observed in M4 (12%) and M5 (9%), which contain the lowest vermiculite content (25% and 20%) and the highest foundry sand content (20% and 25%). Foundry sand’s dense structure and minimal water-retaining characteristics significantly contribute to the reduced water absorption in these mixes^50^. Additionally, the higher compaction and reduced void spaces in these mixes further enhance their resistance to water ingress. These results have practical implications for the application of the mixes in construction. Mixes with higher water absorption, such as M1 and M2, may be suitable for applications where moisture interaction is less critical or where additional coatings can be applied to mitigate water ingress. In contrast, mixes with lower water absorption, such as M4 and M5, are better suited for environments where moisture resistance is essential, such as in outdoor or water-exposed structures^28,49^. A strong inverse relationship is evident between the density and water absorption values across all mixes. Increasing the WFS content in the mixtures resulted in a higher density due to the relatively high specific gravity of WFS compared to vermiculite. Simultaneously, the water requirement decreased as WFS particles reduced void spaces and improved packing density. This behaviour highlights WFS’s ability to enhance compactness in the geopolymer matrix while optimizing water usage. As density increases, the corresponding water absorption decreases, reflecting the densification and reduced porosity of the geopolymer matrix. This relationship emphasizes the role of aggregate selection and mix proportioning in achieving desired performance characteristics in geopolymer composites.

### Compressive strength

**Fig. 5:**
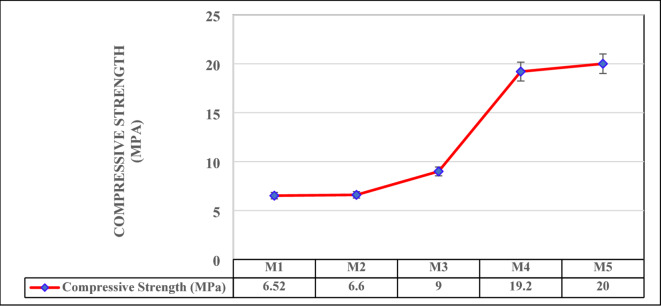
Compressive Strength.

The compressive strength results for the mixes shown in Fig. 5, reveals a clear progression as the proportions of vermiculite and WFS are adjusted, with strength increasing significantly as vermiculite content decreases and WFS content increases. This highlights the influence of material composition on the structural performance of the mixes. M1 and M2, with the highest vermiculite content (40% and 35%, respectively), shows the least compressive strengths at 6.52 MPa and 6.6 MPa, respectively. Lightweight and porous vermiculite material, leads to the lowered mix density and, consequently, lower strength. While these values may limit their application in structural elements, such mixes could still be useful in non-load-bearing applications, such as thermal insulation panels or lightweight wall partitions^28,49^. As the vermiculite content decreases and WFS content increases in M3 (30% vermiculite, 15% foundry sand), the compressive strength rises significantly to 9 MPa. This improvement reflects the increased densification of the mix, as WFS contributes to a more compact and stronger matrix capable of withstanding higher loads.

The trend becomes more pronounced in M4 and M5, where vermiculite content is reduced to 25% and 20%, and WFS content is increased to 20% and 25%, respectively. These mixes achieve the highest compressive strengths, with M4 reaching 19.2 MPa and M5 reaching 20 MPa. The substantial improvement in strength is due to the higher density and reduced porosity of the mixes, as well as the enhanced particle packing and bonding provided by the higher foundry sand content. The results indicate that M4 and M5 are highly suitable for structural applications where strength is a critical requirement, such as in load-bearing walls or flooring systems. In contrast, M1 and M2, while weaker in compressive strength, could be advantageous for lightweight and thermal insulation applications where strength is less critical^28,49^.In conclusion, the compressive strength data underscore the trade-offs between strength and lightweight properties when designing mixes with varying proportions of vermiculite and foundry sand. The compressive strength of mixtures increased with a higher GGBS-to-WFS ratio, owing to enhanced geopolymerization. However, mixtures with higher vermiculite content showed greater weight loss during thermal testing due to its layered structure and moisture retention properties. This trade-off between strength and thermal stability demonstrates the need for balance in material design. These findings allow for material optimization based on specific performance criteria, enabling the selection of mixes tailored for diverse construction needs.

### Flexural strength

**Fig. 6:**
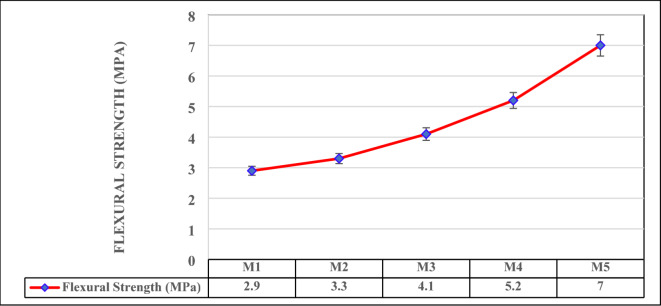
Flexural Strength.

The flexural strength results indicate a consistent improvement in the material’s ability to resist bending stresses as the mix composition transitions from higher vermiculite content to higher WFS content as illustrated in Fig. 6. The trend mirrors the compressive strength results, reinforcing the role of material composition in determining mechanical performance.

M1, with the highest vermiculite content (40%), exhibits the lowest flexural strength at 2.9 MPa. Vermiculite’s lightweight and porous structure reduces the overall matrix density and limits its ability to resist tensile forces during bending. While its flexural performance is lower, this mix could still be appropriate for applications where flexibility and lightweight characteristics are prioritized over strength. In M2, where the vermiculite content is slightly reduced to 35% and foundry sand is increased to 10%, the flexural strength improves to 3.3 MPa. This increase highlights the initial densification of the mix as foundry sand begins to contribute to better particle packing and load distribution within the matrix. M3, with 30% vermiculite and 15% foundry sand, demonstrates a further increase in flexural strength to 4.1 MPa. The gradual replacement of vermiculite with foundry sand enhances the matrix’s structural integrity, allowing it to withstand greater bending forces. The most notable improvements are observed in M4 and M5, with flexural strengths of 5.2 MPa and 7.0 MPa, respectively^48^. These mixes contain the lowest vermiculite content (25% and 20%) and the highest foundry sand content (20% and 25%). Foundry sand, being denser and less porous, significantly enhances the bonding within the matrix, contributing to higher resistance to bending stresses^51,52^. Additionally, the coir fiber additive (5% in all mixes) likely provides reinforcement, bridging cracks and improving the tensile strength of the mixes, with its effect being more pronounced in denser matrices.

The results suggest that M4 and M5 are well-suited for applications where both compressive and flexural strength are critical, such as in flooring, roofing, or structural panels. On the other hand, M1, M2, and M3 may find utility in less demanding structural roles or where lightweight properties and ease of handling are prioritized. In summary, the flexural strength analysis emphasizes the importance of optimizing the balance between vermiculite and foundry sand to achieve the desired mechanical properties. Further, the flexural energy of the samples increased proportionally with density, as denser matrices exhibited higher resistance to cracking. Mixtures with a higher WFS content had improved flexural energy due to better particle packing, which restricted crack propagation under flexural loads. In addition, the role of coir fiber in enhancing tensile behavior also underscores the potential for natural fibers to improve the flexural performance of lightweight and sustainable construction materials.

### Acoustic performance

**Fig. 7:**
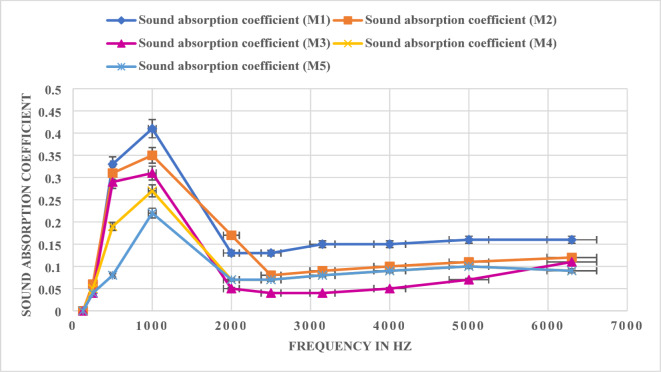
Acoustic Performance.

The sound absorption test results reveal that all mixes exhibit excellent performance across a broad frequency range as shown in Fig. 7. At the lowest frequency of 125 Hz, all mixes show uniform absorption (0.0), indicating that material composition does not significantly impact performance at very low frequencies. At 250 Hz, slight variations emerge, with M2 achieving the highest coefficient (0.06), suggesting that increasing foundry sand content slightly improves absorption in this range. At mid-range frequencies (500 Hz to 1000 Hz), M1 demonstrates the best performance, peaking at 1000 Hz with a coefficient of 0.41. This result indicates that higher vermiculite content enhances absorption in this critical range^53–55^. However, as vermiculite content decreases (M3 to M5), a slight reduction in absorption is observed, with M5 showing the lowest coefficient (0.22) at 1000 Hz. These findings highlight the importance of vermiculite’s porous structure in mid-frequency sound absorption.

At higher frequencies (2000 Hz to 6300 Hz), the absorption coefficients remain relatively consistent across all mixes. M1 and M2, with higher vermiculate content, exhibit slightly better performance at frequencies such as 4000 Hz and 5000 Hz, reaching coefficients of 0.16 and 0.11, respectively. This improvement is likely due to the increased density and rigidity imparted by foundry sand, which enhances high-frequency sound absorption. Mix design demonstrates that vermiculite plays a dominant role in mid-frequency sound absorption, while foundry sand contributes more significantly to high-frequency performance. M2 strikes an optimal balance, exhibiting consistently high absorption across all frequencies. For applications requiring enhanced mid-frequency noise control, M1 and M2 are preferable, whereas M4 and M5 are better suited for environments with high-frequency noise concerns, such as industrial settings^54,55^. From this study, noise absorption coefficients increased with higher vermiculite content due to its porous structure, which efficiently trapped sound waves. Conversely, higher WFS content reduced noise absorption as the denser structure of WFS reflected sound waves rather than absorbing them. These findings provide valuable insights for tailoring material compositions to specific acoustic performance requirements.

### Noise reduction coefficient (NRC)

**Fig. 8:**
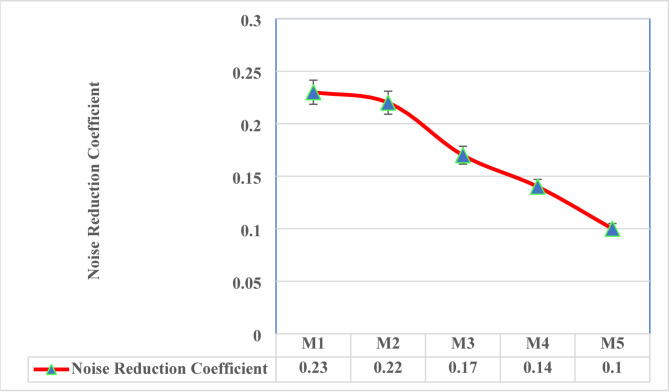
Noise Reduction Coefficient.

The NRC values for the geopolymer mixes demonstrate the acoustic performance of the materials in absorbing sound across different compositions as illustrated in Fig. 8. The values decline gradually from 0.23 (M1) to 0.10 (M5), insisting the impact of the mix design on sound absorption properties. NRC values for the mixes M1 (0.23) and M2 (0.22), demonstrates the highest sound absorption properties. This leads to their higher vermiculite content (40% and 35%), offers a porous structure conducive to sound absorption^18,56^.As lightweight and high porous vermiculite, potentially improves the dissipation of sound energy.

Mixes M3 (0.17) and M4 (0.14) show slightly lower NRC values compared to M1 and M2. As the vermiculite content decreases and WFS content increases, the pore structure of the material becomes denser, reducing its ability to absorb sound. While still effective, these mixes demonstrate a trade-off between mechanical strength and acoustic performance. M5 exhibits the lowest NRC value of 0.10. This result aligns with its composition, where vermiculite content is at its minimum (20%) and foundry sand is at its maximum (25%). The densification of the mix improves strength but compromises the open pore structure necessary for high sound absorption. The NRC values across all mixes indicate that higher vermiculite content contributes to superior noise reduction, while increasing the foundry sand proportion results in a more compact material with reduced acoustic absorption capacity^27,57^. These findings suggest that mixes like M1 and M2 are ideal for applications requiring high noise reduction, such as acoustic panels or sound-insulating barriers. Conversely, M4 and M5 are more suited for environments where strength and durability are prioritized over acoustic performance. The study highlights the versatility of these geopolymer materials, which can be tailored for specific applications by adjusting their mix proportions.

### Thermal performance

**Fig. 9:**
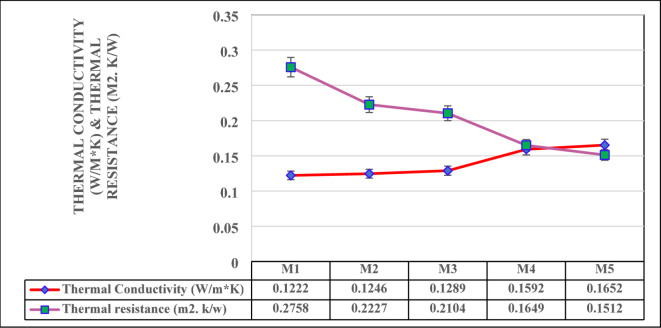
Thermal Conductivity Vs Thermal Resistance.

The thermal performance of the composites is analysed based on their thermal conductivity and thermal resistance, facilitating insights into their heat transfer potential and insulation capabilities is illustrated in Fig. 9. The test results emphasis a clear relationship between the material composition of the mixes and their thermal performance. Thermal Conductivity increases from 0.1222 W/m·K (M1) to 0.1652 W/m·K (M2), highlighting that the gradual addition of vermiculite with foundry sand leads to higher heat transfer rates^58^. Vermiculite, well known porous and low thermal conductivity material, plays a vital role in lowering heat transfer. As the vermiculite content decreases and the denser foundry sand content increases, the thermal conductivity rises because of the higher density and lowered porosity of the mixes. Thermal Resistance is the inverse of thermal conductivity, explores a declining trend from 0.2758 m²·K/W (M1) to 0.1512 m²·K/W(M5). This property is align with the changes in thermal conductivity, as higher conductivity results in lower resistance^59,60^. M1, with the highest vermiculite content (40%), shows the greatest thermal resistance, making it the most effective insulator among the mixes. On the other hand, M5, with the lowest vermiculite content (20%) and highest foundry sand content (25%), exhibits the lowest thermal resistance, indicating reduced insulation capacity.

The results underscore the trade-offs between thermal conductivity and resistance in the mix design. Mixes with higher vermiculite content, such as M1 and M2, are more suitable for applications requiring superior thermal insulation, such as energy-efficient wall panels or insulation layers in construction. Conversely, mixes with higher foundry sand content, such as M4 and M5, are better suited for applications where higher thermal conductivity is desired, such as heat dissipation layers in industrial or high-temperature environments^60,61^. In summary, the thermal characteristics of the mixes highlight the critical role of vermiculite in enhancing insulation performance, while foundry sand increases heat transfer rates. Thermal conductivity values decreased with increased vermiculite content, as its low thermal conductivity acted as an insulator. In contrast, higher WFS content resulted in increased thermal conductivity due to its dense and solid structure, which facilitated heat transfer.

The thermal performance of the geopolymer composites is attributed to the low thermal conductivity of vermiculite and the pore structure within the matrix. The mechanisms underlying heat transfer in these geopolymer mixtures can be explained by considering the contributions of conduction, convection, and radiation. Vermiculite’s layered structure and low density impede heat conduction, while the air pockets within the matrix further disrupt thermal pathways, reducing the overall thermal conductivity. Findings from Berkouche, Amirouche, et al.^62^ reveals that the introduction of lightweight fillers and porous structures effectively minimizes heat transfer by lowering the solid-phase conduction and enhancing the thermal resistance of the composite. Similar to the role of flax fibers in the cited study, the vermiculite in our mixtures provides an additional barrier to heat flow by reinforcing the matrix while maintaining its insulation properties. These findings enable targeted material selection based on specific thermal performance requirements in construction and industrial applications.

### SEM analysis

**Fig. 10:**
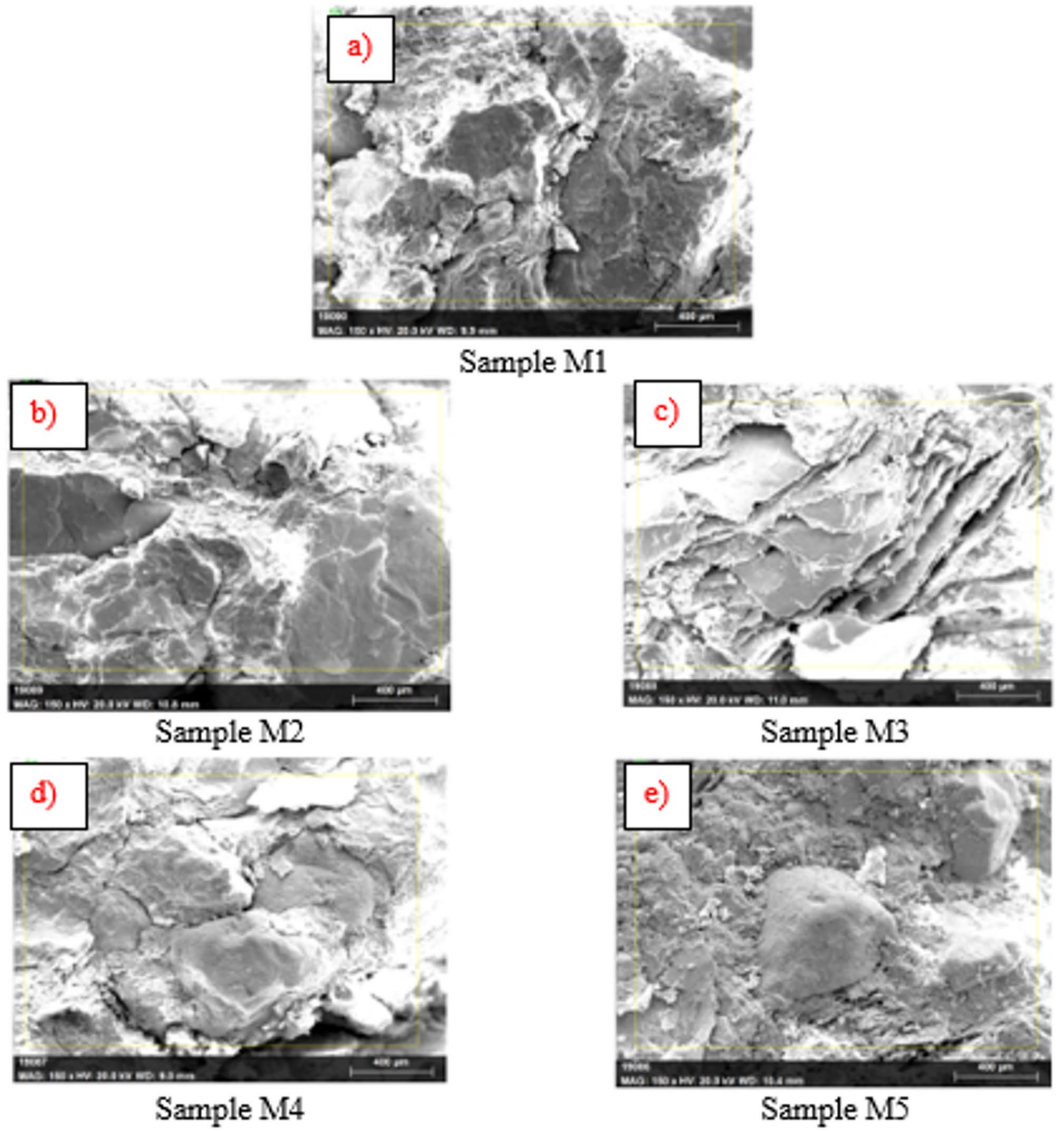
Microscopic analysis (FeSEM) (**a**) Sample M1, (**b**) Sample M2, (**c**) Sample M3,.

The microstructure of sample M1 is categorised by a highly porous composite matrix, mainly because of the high vermiculite content. As it is lightweight and porous material, provides substantial voids and discontinuities within the matrix. Figure 10 demonstrates the microstructure of samples M1 to M5. Further the SEM images demonstrate weak particle bonding among GGBS and vermiculite, with minimum densified hydration products. Coir fibers seem to be embedded and surrounded by microvoids, reducing their load carrying capacity. This porous structure leads to reduction in compressive and flexural strength recorded in the mechanical tests. With reduction in vermiculate and increment in WFS content the sample M2 demonstrates enhanced particle packing with minimum porosity compared to M1. SEM images likely explore better bonding and the coir fibers likely more embedded into the matrix, but the leftover vermiculite content yet leads to some discontinuities and improper hydration^63^. This transitional microstructure in line with the slight enhancement in strength and density when compared to M1.

Further reduction in porosity is observed for sample M3, as appeared in SEM images, with improved compactness of the matrix. The foundry sand particles are uniformly distributed, and loosely packed with GGBS particles and lowering voids. Still the remaining vermiculite content leads to some micro voids that are seen as clustered in particular regions. The interaction between GGBS hydration products and coir fibers is more pronounced, leads to an enhanced load carrying capabilities. This findings are concurrent with mechanical test findings as higher flexural and compressive strength was recorded^64–66^.

d) Sample M4, e) Sample M5.

The SEM images for M4 demonstrates a potential reduction in porosity, with a well-packed composite. The presence of WFS particle packing prevails the microstructure, and hydration products form continuous matrix bond with minimum voids. The influence of vermiculite influence on porosity is minimized, resulting in a more cohesive and denser microstructure. Coir fibers appear firmly anchored in the matrix, with fewer microvoids around them, enhancing tensile strength. These observations are consistent with the mix’s notable improvements in both compressive and flexural strength. The SEM analysis of M5 reveals the most densified matrix among the mixes. The higher foundry sand content ensures minimal voids, and the interaction between GGBS hydration products and filler materials is highly efficient. In contrast, mixtures with more vermiculite exhibited porous structures, which contributed to enhanced thermal and noise insulation but reduced compressive strength. The distribution of hydration products such as calcium silicate hydrate (C-S-H) was more uniform in mixtures with higher GGBS content. Further, in mixtures with increased foundry sand content, hydration product formation was observed to be more localized around WFS particles. This led to the development of a compact geopolymer matrix. However, excessive WFS reduced the availability of reactive GGBS, which slightly impeded the overall geopolymerization process. Optimal WFS ratios promoted balanced hydration, contributing to improved mechanical performance. However, vermiculite’s contribution to porosity is negligible at this low content, resulting in a compact and homogenous structure^64,66^. The coir fibers are well-integrated into the matrix, with minimal pull-out voids or cracks, contributing to the superior mechanical performance observed. The SEM micrographs likely show a continuous and dense structure, explaining the highest compressive and flexural strengths and the lowest water absorption in this mix.

## Comparative analysis on conventional materials

Table 4, emphasis the comparison of bulk density, compressive strength, thermal conductivity, and acoustic performance of various construction materials, in addition with the present study, GGBS-based geopolymer, against other materials derived from fly ash, Portland cement, gypsum, and biomass-based composites. The current study explores a bulk density range of 1302–2032 kg/m³, placing it in a mid-range between lightweight materials like sawdust/geopolymer (334 kg/m³)^37^ and conventional Portland cement board (2150–2550 kg/m³)^67,68^. This promotes its applicability as a medium lightweight material while preserving structural integrity. The increase in bulk density when compared with fly ash-based geopolymers (410–750 kg/m³)^67,68^ and biomass-based composites proposes enhanced packing density and lowered porosity.

The compressive strength of this study increases from 6.52 to 20 MPa, potentially outstanding lightweight composites like wheat straw/geopolymer^37^(0.77 MPa) and sawdust/geopolymer (1.25 MPa). Strength is comparable with gypsum board (4.34–5.36 MPa) and rice husk cement (9.34 MPa) and Portland cement boards (17 MPa), while the geopolymer possesses comparatively high compressive strength, align with its lower bulk density, enables it a competitive substitute for lightweight and medium-load applications^55,69–71^.

While comparing the thermal conductivity of this study (0.1222–0.1652 W/m·K) in some cases it is lower than, other materials such as gypsum board (0.17–0.19 W/m·K) and rice husk cement (0.3554 W/m·K)^67,68^. This emphasis the current ensured as thermally efficient material. It emphasis exceptional insulation properties when compared with Portland cement boards (1.05 W/m·K), enabling it suitable for energy-efficient construction. In addition, its thermal property is comparable with biomass-based materials such as miscanthus/cement (0.0998 W/m·K) and rice husk/geopolymer (0.107 W/m·K)^37,69^.

**Table 4:** Comparison of current study with previous research studies.

Materials	Bulk Density (kg/m3)	Compressive strength (Mpa)	Thermal conductivity (W/m·K)	Acoustic performance	References
**GGBS based Geopolymer**	1302–2032	6.52-20	0.1222–0.1652	0.41	This study
**Flyash based geopolymer**	410–750	0.33–0.83	0.19	0.23	^67,68^
**Portland cement board**	2150–2550	17	1.05	0.108	^67,68^
**Gypsum board**	1540–1900	4.34–5.36	0.17–0.19	0.2	^67,68^
**Rice husk cement**	1311.4	9.34	0.3554	0.05–0.36 at 100–6300	^70^
**Miscanthus/Cement**	NA	5.54	0.0998	0.05–0.73 at 250–1500	^69^
**Sawdust/Gypsum**	1187	6.96	0.204	0.09–0.34 at 125–4000	^55^
**Rice husk/Cement**	745.64	2.85	0.2756	0.005–0.058 at 50–4000	^71^
**Wheat straw/Geopolymer**	340.3	0.77	0.104	0.71 at 200–1600	^37^
**Rice husk/Geopolymer**	387	1.53	0.107	0.57 at 200–1600	^37^
**Sawdust/Geopolymer**	334	1.25	0.089	0.56 at 200–1600	^37^

The acoustic performance of the current study, with a sound absorption coefficient of 0.41, exceeds that of many conventional and alternative materials, such as fly ash-based geopolymers (0.23) and gypsum boards (0.2)^55,69^. However, materials like sawdust/geopolymer and rice husk/geopolymer explores higher sound absorption (0.56–0.71 in specific frequency ranges)^37,71^, the current study facilitates an optimal combination of acoustic and structural performance, enabling it viable for multifunctional applications.

The results demonstrate a balanced interplay between thermal resistance and mechanical performance, governed by the proportions of vermiculite and White Foundry Sand (WFS) in the mixture. Higher vermiculite content enhances thermal resistance due to its excellent insulating properties but reduces mechanical strength owing to its porous structure. Conversely, mixtures with higher WFS content exhibit superior compressive and flexural strength but lower thermal resistance. Among the tested designs, a 30:15 ratio of vermiculite to WFS (Mix M3) emerged as optimal, offering a well-balanced combination of these critical properties for multifunctional applications. This balance highlights the versatility of the proposed mixtures across a range of construction scenarios. Mixtures with higher WFS content, characterized by their enhanced mechanical strength, are suitable for structural components such as partition walls and lightweight load-bearing elements. Meanwhile, mixtures with higher vermiculite content, offering excellent thermal and acoustic insulation, are ideal for nonstructural applications, including insulating wall panels and soundproofing materials. The dual-purpose design significantly broadens the material’s usability, enabling it to meet diverse construction requirements. The improved mixing design underscores the importance of tailoring material composition to meet specific performance demands. By carefully adjusting vermiculite and WFS proportions, the mixtures achieve an optimal blend of thermal, acoustic, and mechanical properties, making them suitable for energy-efficient and sustainable construction practices. Furthermore, the use of industrial byproducts in the design enhances cost-effectiveness and supports a circular economy, contributing to both environmental sustainability and modern construction needs.

Future research could focus on enhancing the thermal stability of the proposed mixtures by incorporating advanced nanomaterials, such as aerogels or silica nanoparticles, to improve insulation properties without compromising mechanical strength. Additionally, studying the long-term durability of these mixtures under extreme conditions, including freeze-thaw cycles, high humidity, and temperature fluctuations, is essential to assess their performance in harsh environments. Optimizing alkaline activation by exploring alternative activators and activation methods could further refine the geopolymerization process, tailoring the material properties for specific applications. Incorporating natural or synthetic fibers holds potential for improving toughness, crack resistance, and ductility while maintaining thermal resistance. Finally, conducting comprehensive life cycle assessments would help quantify the environmental benefits of the proposed mixtures, demonstrating their sustainability compared to conventional construction materials^72,73^.

The current study explores a novel mix for balance of mechanical, thermal, and acoustic properties. Further, compressive strength and thermal performance of this study are outstanding with many biomass-based composites, while its acoustic property is excellent to conventional materials such as gypsum and cement boards. When compared to fly ash-based geopolymers, the current study composites promote strength and density, enabling it a more demanding material for a wide range of construction applications.

## Conclusion

The geopolymer composites analysed in this study exhibit potential for sustainable and energy efficient multifunctional applications construction industry The test findings explore that by modifying the mix combination of vermiculite and WFS, the composite can attain a balance between mechanical, thermal, and acoustic performance. Key technical finding of this study is summarized below:

- Compressive strength varies from 6.52 MPa to 20.0 MPa, wherein strength improvement subjected to WFS increment, facilitated to enhanced particle packing and composite densification. Flexural strength enhances from 2.9 MPa to 7.0 MPa as increment in WFS content, emphasizing its role in promoting toughness and crack resistance. These test findings concur with geopolymerisation densification theories.
- Thermal conductivity ranges from 0.1222 W/m·K to 0.1652 W/m·K and thermal resistance ranges from 0.1512 m²·K/W to 0.2758 m²·K/W, exhibiting exceptional insulation potential, particularly in vermiculite-rich composites. The vermiculate porous structure efficiently captures air, lowering heat transfer. The sound absorption coefficient, maximum at 0.41 at 1000 Hz, emphasis significant acoustic performance, enabling these mixes suitable for soundproofing in lightweight construction application.
- The progression towards lightweight (1302 kg/m³) to medium-dense materials (2032 kg/m³) is attained by varying the mix combinations, promoting material design flexibility for specific applications. Further water absorption reduces from 23 to 9% with lowered porosity in foundry sand-rice composite, improving durability and resistance against moisture-related deterioration.

From this study vermiculite-rich composites having high thermal insulation and acoustic absorption, enabling them highly applicable for energy-efficient, noise-reducing building applications. These properties are led to vermiculite’s low thermal conductivity and its potential to grasp air within its porous structure, which has been validated by several studies in the field. In addition, foundry sand-rich mixes, offer improved mechanical performance, facilitating significant importance for structural applications. The compressive and flexural strength improvement, linked with lowered water absorption, promotes the use of these mixes in environments where material durability and resistance to degradation are predominant.

While the study demonstrates significant advancements in the development of geopolymer composites incorporating GGBS, WFS, and vermiculite for enhanced thermal and mechanical performance, certain limitations should be acknowledged. First, the long-term durability of these composites under extreme environmental conditions, such as freeze-thaw cycles and high humidity, was not investigated. Second, the use of specific industrial byproducts may introduce variability in material properties due to potential differences in their chemical compositions across different sources. Third, while the thermal and noise insulation properties were evaluated, the practical performance of these composites in real-world applications, such as geopolymer wall panels, requires further validation. Future research should address these limitations to further refine the applicability and scalability of geopolymer composites in construction.

## Data Availability

The datasets used and/or analysed during the current study available from the corresponding author on reasonable request.

## References

[1] Sen, B., Tam, N., Maharjan, R., Maharjan, A. K. & Talukdar, G. The shift towards green construction: A review of environmental management strategies and sustainable materials in developed countries. *Trop. Environ. Biol. Technol.* **2**, 80–92 (2024).

[2] Miller, S. A., Juenger, M., Kurtis, K. E. & Weiss, J. Cement and alternatives in the anthropocene. *Annu. Rev. Environ. Resour.* **49**(1), 309–335 (2024).

[3] Sbahieh, S., Serdar, M. Z. & Al-Ghamdi, S. G. Decarbonization strategies of building materials used in the construction industry, Mater. Today Proc. (2023).

[4] Nanthini, M., Ganesan, R. & Jaganathan, V. Studies on alkaline activator, manufacturing methods and mechanical properties of geopolymer Concrete-A. *J. Environ. Nanotechnol*. **13**, 52–72 (2024).

[5] Kalinowska-Wichrowska, K. et al. Geopolymer Composites with Recycled Binders, in: Int. Congr. Polym. Concr., Springer, : pp. 212–219. (2023).

[6] Zhang, Z. et al. The influence of fly Ash and slag on the mechanical properties of geopolymer concrete. *Buildings* **14**, 2720 (2024).

[7] Liew, K. M., Sojobi, A. O. & Zhang, L. W. Green concrete: prospects and challenges. *Constr. Build. Mater.* **156**, 1063–1095 (2017).

[8] Almutairi, A. L., Tayeh, B. A., Adesina, A., Isleem, H. F. & Zeyad, A. M. Potential applications of geopolymer concrete in construction: A review, case stud. *Constr. Mater.* **15**, e00733 (2021).

[9] Shilar, F. A., Ganachari, S. V. & Patil, V. B. Investigation of the effect of granite waste powder as a binder for different molarity of geopolymer concrete on fresh and mechanical properties. *Mater. Lett.* **309**, 131302 (2022).

[10] Shilar, F. A. et al. Evaluation of the effect of granite waste powder by varying the molarity of activator on the mechanical properties of ground granulated blast-furnace slag-based geopolymer concrete. *Polym. (Basel)*. **14**, 306 (2022). 10.3390/polym14020306 PMC8779299 35054712

[11] Shilar, F. A. et al. Assessment of destructive and nondestructive analysis for GGBS based geopolymer concrete and its statistical analysis. *Polym. (Basel)*. **14**, 3132 (2022). 10.3390/polym14153132 PMC9371249 35956647

[12] Van Lam, T. & Nguyen, M. H. Incorporating industrial By-Products into geopolymer mortar: effects on strength and durability. *Mater. (Basel)*. **16**, 4406 (2023). 10.3390/ma16124406 PMC10301944 37374588

[13] Adhitya, B. B., Saggaff, A., Saloma, S. & Hanafiah, H. A review of geopolymers-based artificial aggregates technology developed using waste materials. *Civ. Eng. Archit.* **12**, 1338–1349 (2024).

[14] Thapa, S., Debnath, S., Kulkarni, S., Solanki, H. & Nath, S. Mechanical properties of geopolymer concrete incorporating supplementary cementitious materials as binding agents. *Discov Civ. Eng.* **1**, 62 (2024).

[15] Nguyen, Q. D. & Castel, A. Developing geopolymer concrete by using ferronickel slag and ground-granulated blast-furnace slag. *Ceramics* **6**, 1861–1878 (2023).

[16] Moujoud, Z. et al. Geopolymer composites reinforced with natural fibers: A review of recent advances in processing and properties. *Constr. Build. Mater.* **388**, 131666 (2023).

[17] Villaquirán-Caicedo, M. A., Perea, V. N., Ruiz, J. E. & de Gutiérrez, R. M. Mechanical, physical and thermoacoustic properties of lightweight composite geopolymers. *Ing. Y Compet.* **24**, 1 (2022).

[18] Aly, N. M., Seddeq, H. S., Elnagar, K. & Hamouda, T. Acoustic and thermal performance of sustainable fiber reinforced thermoplastic composite panels for insulation in buildings. *J. Build. Eng.* **40**, 102747 (2021).

[19] Sambucci, M., Sibai, A. & Valente, M. Recent advances in geopolymer technology. A potential eco-friendly solution in the construction materials industry: A review. *J. Compos. Sci.* **5**, 109 (2021).

[20] Chen, Y. X., Klima, K. M., Brouwers, H. J. H. & Yu, Q. Effect of silica aerogel on thermal insulation and acoustic absorption of geopolymer foam composites: the role of aerogel particle size. *Compos. Part. B Eng.* **242**, 110048 (2022).

[21] Zhang, Z., Provis, J. L., Reid, A. & Wang, H. Mechanical, thermal insulation, thermal resistance and acoustic absorption properties of geopolymer foam concrete. *Cem. Concr Compos.* **62**, 97–105 (2015).

[22] Lawanwadeekul, S., Jun-On, N., Kongthavorn, P., Sangkas, T. & Daothong, S. Chemical-free thermal-acoustic panels from agricultural waste for sustainable Building materials. *Clean. Mater.* **12**, 100245 (2024).

[23] Ahmad, J. et al. A comprehensive review on the ground granulated blast furnace slag (GGBS) in concrete production. *Sustainability* **14**, 8783 (2022).

[24] Gencel, O., Gholampour, A., Tokay, H. & Ozbakkaloglu, T. Replacement of natural sand with expanded vermiculite in fly ash-based geopolymer mortars. *Appl. Sci.* **11**, 1917 (2021).

[25] Kanagaraj, B., Anand, N., Alengaram, U. J., Raj, R. S. & Kiran, T. Exemplification of sustainable sodium silicate waste sediments as coarse aggregates in the performance evaluation of geopolymer concrete. *Constr. Build. Mater.* **330**, 127135 (2022).

[26] Kumar, V. V., Bhikshma, V. & Prasad, B. V. Investigation on high-strength geopolymer concrete with UFGGBS and recycled coarse aggregate. *Libr. Prog Int.* **44**, 695–706 (2024).

[27] Narattha, C., Wattanasiriwech, S. & Wattanasiriwech, D. Sustainable, multifunctional fly Ash geopolymer composite with rice husk aggregates for improved acoustic, hygric, and thermal performance. *Constr. Build. Mater.* **445**, 137743 (2024).

[28] Ganasen, N., Bahrami, A. & Loganathan, K. Experimental investigation and optimization of lightweight concrete bricks developed using vermiculite. *Front. Mater.* **10**, 1117138 (2023).

[29] Lopez, A., Bazaez, R., Leiva, G., Loyola, R. & Gómez, M. Experimental study of in-plane flexural behavior of screen-grid insulated concrete form rectangular and T-shaped walls. *Eng. Struct.* **247**, 113128 (2021).

[30] Kanagaraj, B., Kiran, T., N, A. & Al Jabri, K. Development and strength assessment of eco-friendly geopolymer concrete made with natural and recycled aggregates. *Constr. Innov.* **23**, 524–545 (2023).

[31] Sunarsih, E. S., As’ ad, S., Mohd, A. R., Sam, S. & Kristiawan The effect of alkali activator to binder ratio on workability, density, and compressive strength of fly Ash-Slag based geopolymer mortar. *Nano Hybrids Compos.* **45**, 121–127 (2024).

[32] Yao, Z., Luo, L., Qin, Y., Cheng, J. & Qu, C. Research on mix design and mechanical performances of MK-GGBFS based geopolymer pastes using central composite design method. *Sci. Rep.* **14**, 9101 (2024). 38643269 10.1038/s41598-024-59872-0 PMC11032377

[33] Buczkowska, K. E. et al. Maximizing performance of geopolymer mortar: optimizing basalt and carbon Fiber content composition. *J. Nat. Fibers*. **21**, 2293047 (2024).

[34] Amaludin, A. E., Asrah, H., Mohamad, H. M., Amaludin, N. A. & Amaludin, H. Z. Fresh and hardened properties of Alkali-Activated POFA-GGBFS pastes cured in ambient Temperature–An initial mix design. *J. Adv. Res. Appl. Mech.* **125**, 103–115 (2024).

[35] Wang, Z. et al. Investigation and utilization of Alkali-Activated grouting materials incorporating engineering waste soil and fly ash/slag. *Appl. Sci.* **14**, 4915 (2024).

[36] Faruqui, A. N., Akter, M. T., Biswas, R. & Sheikh, M. R. K. Coir powder-reinforced epoxy resin composites: fabrication and characteristics analysis. *J. Polym. Sci. Eng.* **7**, 7394 (2024).

[37] Wang, S. et al. Experimental study on durability and acoustic absorption performance of biomass geopolymer-based insulation materials. *Constr. Build. Mater.* **361**, 129575 (2022).

[38] Liu, L. et al. Experimental physical properties of an eco-friendly bio-insulation material based on wheat straw for buildings. *Energy Build.* **201**, 19–36 (2019).

[39] Wang, S., Li, H., Zou, S. & Zhang, G. Experimental research on a feasible rice husk/geopolymer foam Building insulation material. *Energy Build.* **226**, 110358 (2020).

[40] Zou, S. et al. Experimental research on an innovative sawdust biomass-based insulation material for buildings. *J. Clean. Prod.* **260**, 121029 (2020).

[41] Feng, X., Liu, N. & Lu, X. Investigation of un-calcined coal gangue together with ground granulated blast furnace slag and fly Ash to ambient-curing production high-strength geopolymer. *J. Mater. Res. Technol.* **25**, 3985–3997 (2023).

[42] Standard, A. Standard test method for sound absorption and sound absorption coefficients by the reverberation room method, C423-90a (1990).

[43] Abdalla, J. A. et al. A comprehensive review on the use of natural fibers in cement/geopolymer concrete: A step towards sustainability, case stud. *Constr. Mater.* **19**, e02244 (2023).

[44] A.S & for, T. *And M.C.C.-1 on Cement, Standard Test Method for Compressive Strength of Hydraulic Cement Mortars (Using 2-in. Or [50-mm] Cube Specimens)* (ASTM International, 2013).

[45] Salim, S. G. R. Thermal conductivity measurements using the transient hot-wire method: a review. *Meas. Sci. Technol.* **33**, 125022 (2022).

[46] Xu, L., Ding, Y. & Du, C. Thermal performance assessment of exterior Building walls under intermittent air-conditioning operation in china’s hot summer and cold winter zone. *J. Build. Eng.* **95**, 110204 (2024).

[47] I.S.O. 10534-2, Acoustics—Determination of Sound Absorption Coefficient and Impedance in Impedance tubes—Part 2: Transfer-function method (1998).

[48] Siddique, R. & Singh, G. Utilization of waste foundry sand (WFS) in concrete manufacturing. *Resour. Conserv. Recycl*. **55**, 885–892 (2011).

[49] Jssem, M. & Fawzi, N. M. Effect of expanded perlite aggregate and silica fume on some properties of lightweight concrete. *J. Eng.* **30**, 172–185 (2024).

[50] Paul, A., Dey, P., Das, A. & Debnath, A. Manufacturing of sand based aerated concrete bricks with fibre Reinforcement-An experimental study. *Educ. Adm. Theory Pract.* **30**, 3440–3455 (2024).

[51] Zheng, R. et al. Feasibility of waste foundry sand in high-strength self-compacting concrete and the effects of elevated temperatures. *Constr. Build. Mater.* **402**, 133075 (2023).

[52] Kumar, R. S., Devarajan, P., Kumar, A. S., Kadavan, A. S. & Rajesh, R. Performance Assessment and Engineering behaviour of Cement Concrete with partially Replaced Foundry Sand as Fine Aggregate, in: IOP Conf. Ser. Earth Environ. Sci., IOP Publishing, : p. 12007. (2023).

[53] Ganesh, A. C., Muthukannan, M., Aakassh, S. & Subramanaian, B. Energy efficient production of geopolymer bricks using industrial waste, in: IOP Conf. Ser. Mater. Sci. Eng., IOP Publishing, : p. 12154. (2020).

[54] Liu, C. et al. Energy retrofitting assessment of public Building envelopes in china’s hot summer and cold winter climate region. *Buildings* **12**, 1866 (2022).

[55] Pedreño-Rojas, M. A., Morales-Conde, M. J., Pérez-Gálvez, F. & Rodríguez-Liñán, C. Eco-efficient acoustic and thermal conditioning using false ceiling plates made from plaster and wood waste. *J. Clean. Prod.* **166**, 690–705 (2017).

[56] Belakroum, R. et al. Hygric buffer and acoustic absorption of new Building insulation materials based on date palm fibers. *J. Build. Eng.* **12**, 132–139 (2017).

[57] Moussa, T. et al. Spent coffee grounds as Building material for non-load-bearing structures. *Mater. (Basel)*. **15**, 1689 (2022). 10.3390/ma15051689 PMC8911109 35268920

[58] Peng, X., Li, H. & Hu, Y. Preparation of metakaolin-fly Ash cenosphere based geopolymer matrices for passive fire protection. *J. Mater. Res. Technol.* **23**, 604–610 (2023).

[59] Bourzik, O. et al. Life cycle assessment and thermophysical properties of a fly ash-based geopolymer containing drinking water treatment sludge. *Environ. Sci. Pollut Res.* **30**, 118989–119000 (2023). 10.1007/s11356-023-30736-w 37923887

[60] Mahapatra, D., Madav, V. & Setty, A. B. T. P. Mechanical and dynamic thermal performance evaluation of rice husk blended cement plaster when used with different bricks. *J. Build. Eng.* **82**, 108120 (2024).

[61] Chabannes, M., Bénézet, J. C., Clerc, L. & Garcia-Diaz, E. Use of Raw rice husk as natural aggregate in a lightweight insulating concrete: an innovative application. *Constr. Build. Mater.* **70**, 428–438 (2014).

[62] Berkouche, A. et al. Experimental optimization of Low-Carbon cellular foam geopolymers incorporating crushed stone sand and flax Fiber using central composite design. *Iran. J. Sci. Technol. Trans. Civ. Eng.* **48**, 1–21 (2024).

[63] Chabannes, M., Garcia-Diaz, E., Clerc, L., Bénézet, J. C. & Becquart, F. *Lime Hemp and Rice husk-based Concretes for Building Envelopes* (Springer, 2018).

[64] Tajunnisa, Y. et al. Effect of GGBFS and micro-silica on mechanical properties, shrinkage and microstructure of alkali-activated fly Ash mortar. *GEOMATE J.* **13**, 87–94 (2017).

[65] de Matos, P. R. et al. Self-compacting mortars produced with fine fraction of calcined waste foundry sand (WFS) as alternative filler: Fresh-state, hydration and hardened-state properties. *J. Clean. Prod.* **252**, 119871 (2020).

[66] Zhang, H. Y. & He, H. H. Characterization of microstructure of FA ceramic brick using SEM. *Adv. Mater. Res.* **849**, 283–286 (2014).

[67] Jayajothi, P., Kumutha, R. & Vijai, K. Properties of fly Ash and GGBS based geopolymeric binder. *Chem. Sci. Rev. Lett.* **2**, 470–479 (2014).

[68] Liu, X., Hu, C. & Chu, L. Microstructure, compressive strength and sound insulation property of fly ash-based geopolymeric foams with silica fume as foaming agent. *Mater. (Basel)*. **13**, 3215 (2020). 10.3390/ma13143215 PMC7412515 32707705

[69] Gao, H. et al. A bifunctional hierarchical porous kaolinite geopolymer with good performance in thermal and sound insulation. *Constr. Build. Mater.* **251**, 118888 (2020).

[70] Marques, B. et al. Rice husk cement-based composites for acoustic barriers and thermal insulating layers. *J. Build. Eng.* **39**, 102297 (2021).

[71] Pachla, E. C., Silva, D. B., Stein, K. J., Marangon, E. & Chong, W. Sustainable application of rice husk and rice straw in cellular concrete composites. *Constr. Build. Mater.* **283**, 122770 (2021).

[72] Khater, H. M. & Gharieb, M. Enhancing physico-mechanical properties and thermal stability of geopolymer composites through nano-material incorporation. *Discov Appl. Sci.* **6**, 206 (2024).

[73] Xu, Z. et al. High temperature thermal insulation ceramic aerogels fabricated from ZrC nanofibers welded with carbon nanoparticles, ACS appl. *Nano Mater.* **7**, 10046–10055 (2024).

